# Interleukin-28B dampens airway inflammation through up-regulation of natural killer cell-derived IFN-γ

**DOI:** 10.1038/s41598-017-03856-w

**Published:** 2017-06-15

**Authors:** Bailing Yan, Feng Chen, Lijun Xu, Yanshi Wang, Xuefu Wang

**Affiliations:** 1 Department of Emergency, The First Hospital of Jilin University, Changchun, 130021 China; 2 Dermatology Department, China-Japan Union Hospital of Jilin University, Changchun, 130033 China; 3 Department of Respiratory Medicine, The First Hospital of Jilin University, Changchun, 130021 China; 4 Institute of Immunology, School of Life Sciences, University of Science and Technology of China, Hefei, Anhui 230027 China; 5 School of Pharmacology, Anhui Medical University, Hefei, Anhui 230032 China

## Abstract

Interleukin-28A (IL-28A) modulates CD11c+ dendritic cell (DC) function and promotes type 1T helper (Th1) differentiation, thus suppressing allergic airway diseases. However, the function of the IL-28A isoform IL-28B in these diseases remains largely unknown. In this study, we revealed a novel role of IL-28B in inducing type 1 immunity and protecting against ovalbumin (OVA)-induced allergic asthma in mice. IL-28B overexpression in wild-type mice promoted natural killer (NK) cell polarization in the lung, leading to the increased number of interferon (IFN)-γ-producing NK1 cells as well as Th1 differentiation. Importantly, IL-28B overexpression had no protective effect on OVA-induced asthma in IFN-γ-knockout (IFN-γ−/−) mice. These results demonstrate that IL-28B ameliorates experimental allergic asthma via enhancing NK cell polarization, which might be useful for prevention and treatment of allergic asthma.

## Introduction

Asthma is a common chronic inflammatory disorder of the airways characterized by irreversible airway obstruction. It is estimated that up to 300 million people worldwide suffer from asthma^1^. Asthma development is a complex process and involves epithelial remodeling and subsequent epithelial injury and repair that are attributable to a variety of factors, including inflammatory cells and inflammatory mediators^2^. Due to these inflammatory stimuli, inappropriate Th2-mediated immune responses are induced, resulting in production of IgE, the infiltration of lymphocytes and eosinophils, mucus overproduction, and airway hyper-reactivity (AHR)^1^.

NK cells, a component of the innate immunity, are more abundant in the lung than in other organs, such as the liver and spleen^3–5^. Alike T cells, NK cells can be divided into different subsets such as NK1, NK2, NK17 or NKreg cells according to their cytokine production including IFN-γ, IL-4, IL-17, and IL-10. Based on the profile of cytokine production, NK cells are divided into different functional subsets: INF-γ-producing NK1 cells, IL-4-producing NK2 cells, IL-17-producing NK17 cells, and IL-10-producing NKreg cells^6–9^. IFN-γ production from NK cells can polarize CD4+ T-cells toward a Th1 phenotype^10^, whereas NK2 cells are associated with asthma exacerbation. As a result, the immunologic interventions preventing NK2 bias might benefit patients with asthma^11, 12^.

*In vivo*, micro-environmental factors can condition the generation and function of distinct NK cell subsets^13^. It was documented that IL-4^+^ NK cell frequency was increased in patients with asthma compared to healthy individuals. In contrast, IFN-γ^+^ NK cell frequency was decreased. Furthermore, successful treatment of the asthmatics correlates with a reversal of the shift toward NK2 cells, suggesting that an NK2 cell subset is involved in the pathogenesis of asthma^11^. Consistent with these findings, NK cells from allergic individuals may lead to a significant increase in IL-5- and IL-13-producing NK2 subsets which are involved in the development of allergic inflammation^14^.

IL-28, a new family of cytokines which was discovered in 2003, is described as λ-interferons or type III interferons (IFN) including IL-28A, IL-28B and IL-29, whereas mouse λ-interferons have only two members (IL-28A and IL-28B)^15^. The mechanism in which IL-28A inhibits airway inflammation has been reported in the previous study^16^. However, the mechanism of action of IL-28B in airway inflammation has never been explored. IL-28R is a heterodimeric receptor consisting of IL-28Rα and IL-10Rβ^17^. Among them, IL-10Rβ is ubiquitously expressed and shared with all other IL-10 family members. In contrast, IL-28Rα is expressed on epithelium-derived cells like hepatocytes and myeloid lineage cells, such as dendritic cells (DCs) and macrophages^18, 19^, and are responsible for ligand specificity. Recently, human and mouse NK cells were found to also express IL-28Rα^20^. Previous study demonstrated that IL-28A suppresses allergic airway disease through modulating lung DC function and promoting Th1 differentiation^16^. In the present study, we found that IL-28B could increase the frequency and number of NK cells in OVA-challenged mouse lung, which prompts us to investigate the role of IL-28B in OVA-induced airway inflammation and the underlying mechanisms. As we demonstrated, IL-28B promoted NK1 cell polarization *in vivo* and suppressed OVA-induced allergic asthma in mice, suggesting NK cells are potential immunotherapeutic agents.

## Materials and Methods

### Animals

All experimental protocols were approved by the Institutional Ethics Committee for Animal Use in Research of University of Science and Technology of China (USTC; Hefei, China) and the methods were carried out in accordance with Animal Care guidelines of USTC. Male C57BL/6 mice aged 6–8 weeks were purchased from Shanghai SLAC Laboratory Animal center, Chinese Academy Science (Shanghai, China). IFN-γ−/− mice on a C57BL/6 genetic background were kindly provided by Dr. Shaobo Su (Tongji University School of Medicine, Shanghai, China). All mice were housed in micro-isolator cages under humidity- and temperature-controlled specific pathogen-free condition in the animal facility of the School of Life of USTC.

### Antibodies and recombinant plive vectors

AsGM1 Antibody was purchased from Wako Co., Ltd. (Tokyo, Japan). The plive vector is a kind of liver-specific transgene expression vector which utilizes a chimeric promoter composed of the minimal mouse albumin promoter and mouse alpha fetoprotein enhancer II. Recombinant plive vector expressing IL-28B (plive-IL-28B) was kindly provided by Dr. Yanshi Wang at USTC, and was amplified using the EndoFree Maxi plasmid kit (Macherey-Nagel, Duren, Germany). OVA was purchased from Sigma-Aldrich (St. Louis, MO, USA). Aluminum adjuvant was purchased from Thermo Fisher Scientific (Rockford, IL, USA).

### Cytokine Detection

IL-28B were detected using mouse IL-28 platinum ELISA Kit (eBioscience, CA, USA) according to the manufacturer’s instructions.

### Measurement of IgE in serum

The mouse sera were isolated and frozen at −80 °C before use. The concentrations of total IgE in serum were determined using the Mouse IgE ELISA kits (Dakewe Biotech Co., Ltd., Shenzhen, China), following the manufacturer’s instructions.

### Hydrodynamic injection

The plive-IL-28B was purified using the EndoFree Maxi plasmid kit (Macherey-Nagel, Duren, Germany). 20 mg of purified plive-IL-28B dissolved in PBS in a volume equivalent to 8% of the mouse body weight was injected via tail veins within 5 seconds on day 1 as indicated in the experimental protocol. The same dose of null plive-vector and equivalent volume of PBS were given as control respectively.

### Allergen sensitization and challenge protocol and treatment regimens

All mice were sensitized with two intraperitoneal injections on days 0 and 7 of 100 μg OVA (Grade V; Sigma-Aldrich, St. Louis, MO, USA) complexed with 50 μL adjuvant aluminum hydroxide (Thermo Fisher Scientific, Rockford, IL, USA). On days 14, 15 and 16, mice were administered intranasally with 50 μg OVA in a volume of 50 μL.

### *In vivo* depletion of NK cells

To deplete NK cells^21^, mice were administered anti-asialo GM1 Ab (AsGM1) (50 μL/mouse, i.v.; Wako Pure Chemical Industries, Ltd., Japan)^22^ or control IgG (Rabbit) 1 day before OVA challenges indicated in the experimental protocol.

### Collection and analysis of the BALF

24 h after the last OVA challenge, BALF was obtained by rinsing lungs followed by centrifugation with 0.8 mL PBS at 1500 rpm for 5 min. The supernatants were analyzed by ELISA, and the pellets were washed three times with 1.0 mL PBS followed by centrifugation at 1500 rpm for additional 5 min. All cell pellets were suspended in 1.0 mL PBS.

### Isolation of lymphocytes

Mice were anesthetized, then lungs were collected, excised and minced, followed digestion for 60 min at 37° with RPMI-1640 containing 0.1% collagenase IV (Sigma, St Louis, MO) and 5% fetal calf serum. The large pieces of lung were removed by filtration through gauze. The residual RBCs were lysed with RBC lysis buffer (Biolegend, San Diego, CA, USA). The resultant single cell suspensions were washed three times with ice cold PBS. Cell numbers were determined by trypan blue exclusion method.

### Histological analysis

Lung tissues were fixed in 10% (vol/vol) neutral buffered formalin and embedded in paraffin. Sections (5 µm) were stained with hematoxylin/eosin (H&E) or periodic acid–Schiff’s (PAS) solution (Sigma-Aldrich, St. Louis, MO, USA). Histopathologic analysis of inflammatory cells in H&E stained lung sections from five mice was performed in a blinded fashion using a semi-quantitative scoring system as previously described^16, 23^. Peribronchiolar inflammation were scored as follows: 0, normal; 1, few cells; 2, a ring of inflammatory cells one cell layer deep; 3, a ring of inflammatory cells two to four cells deep; and 4, a ring of inflammatory cells of more than four cells deep. Morphometric analysis of PAS stained sections was performed by quantifying PAS pixels per μm length using the Image J software. At least six randomly selected fields in each section were assessed blindly by an expert pathologist.

### Flow cytometric analysis

After the blockade of the Fc receptor by rat serum, single-cell suspensions were incubated with the fluorescently labelled monoclonal antibodies at 4° for 30 min in PBS containing 0.1% sodium azide and 1% bovine serum albumin, and then washed twice followed by fixation, permeabilization, and staining with phycoerythrin-conjugated anti-IFN-γ or isotype control. Samples were analyzed by flow cytometry (LSR II; BD Biosciences, San Diego, USA) using FLOWJO 7.6.1 software. The anti-mouse monoclonal antibodies used for flow cytometry were as follows: NK1.1, CD45, CD3, IFN-γ, CD4, CD19, γδTCR, F4/80, CD11c, CD11b, SiglecF, MHC-II, and CD8. All antibodies and isotype controls were purchased form BD Biosciences (Franklin Lakes, NJ, USA), R&D Systems (Minneapolis, MN, USA), or eBioscience (San Diego, CA, USA). Cell phenotypes were identified as follows: Eosinophils, CD45+SiglecF+CD11b+CD11c−; Neutrophils, CD45+CD11b+Ly6G(high); Lymphocytes, CD45+CD3+/CD19+/NK1.1+; Macrophages, CD45+F4/80(high)CD11c(high)CD11b−; Dendritic cells CD45+MHCII+CD11c(high) F4/80−. To calculate the absolute numbers cells, we used the formulas: absolute NK cell numbers = total lymphocytes × (% of NK1.1e+ cells). Total eosinophils = Total leukocytes × (% of SiglecF+ cells) × (% of CD11b+CD11c− cells); Neutrophils = Total leukocytes × (% of CD11b+Ly6G (high) cells); Lymphocytes = Total leukocytes × (% of CD3+/CD19+/NK1.1+ cells); Macrophages = Total leukocytes × (% of CD11c(high)CD11b− cells) × % of F4/80(high)cells; Dendritic cells = Total leukocytes ×  (% of MHCII+CD11c(high) cells)  × (% of F4/80− cells).

### Airway hyper-responsiveness

In response to different concentrations of methacholine, the airway resistance was measured invasively in pentobarbital sodium-anesthetized mice 24 h after the last OVA challenge as previously described^24^, using a respiratory function instrument (AniRes2005, BioMedical Supply Co., Ltd., Beijing, China).

### Statistical analysis

All data were expressed as mean ± SD and analyzed using the Student’s two-tailed *t*-test or one-way analysis of variance (ANOVA) test followed by LSD’s multiple comparison test. A value of *P* < 0.05 was considered statistically significant.

## Results

### Overexpression of IL-28B inhibited OVA-induced allergic asthma in mice

**Figure 1:**
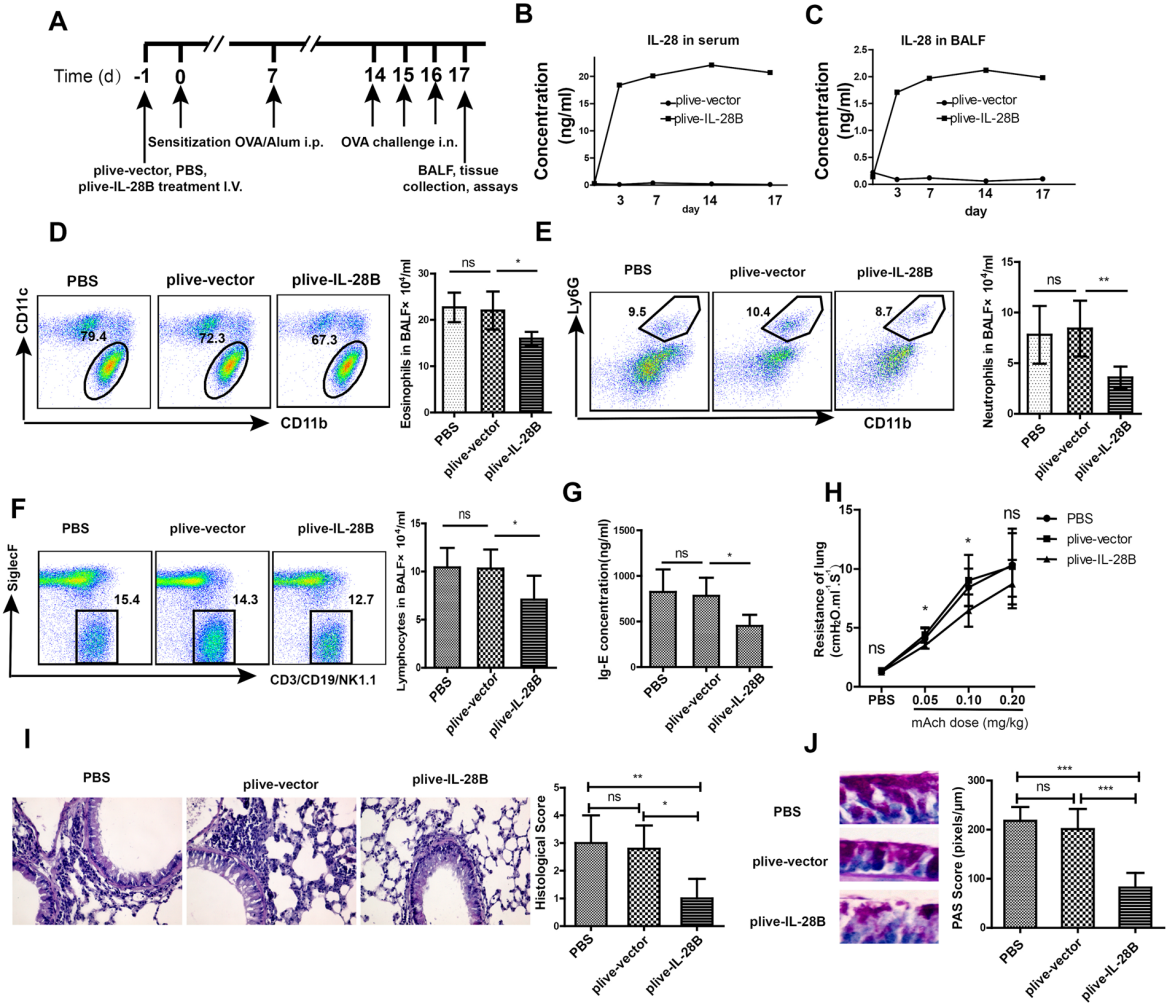
IL-28B suppressed OVA-induced airway inflammation. (**A**) Schematic timeline for OVA induction of asthma and plive-IL-28B injection. (**B**) and (**C**) Time-course expression of IL-28B in serum (**B**) and BALF (**C**). (**D–F**) Flow cytometry and cell counting assays for abundance and numbers of eosinophils (**D**) neutrophils (**E**) and lymphocytes (**F**) in BALF. (**G**) The inhibitory effect of plive-IL-28B on IgE production. (**H**) The effect of plive-IL-28B on the airway resistance in the mouse lung. (**I**) Histological assessment of lung inflammation in PBS-, plive-vector- or plive-IL28B- treated asthmatic mice. (**J**) PAS staining for mucus production in lung epithelium of PBS-, plive-vector- or plive-IL28B- treated asthmatic mice. PBS is phosphate buffer saline. OVA is ovalbumin. Alum is aluminum. BALF is bronchoalveolar lavage fluid. I.V. is intravenous injection. i.p. is intraperitoneal injection. i.n. is nasal inhalation. mAch is muscarinic acetylcholine. PAS is Periodic Acid-Schiff stain. H&E is hematoxylin-eosin staining. *p < 0.05; **p < 0.01; ***p < 0.001; ns: non-significant (*n* = 5 mice per group). Results shown are representative of three independent experiments.

IL-28A has been shown to inhibit OVA-induced allergic airway diseases^24^. However, the role of IL-28B in these diseases has never been investigated. In this study, we overexpressed IL-28B in mice by hydrodynamic tail vein injections of 20 µg plasmids plive-IL-28B or plive-vector and PBS one day prior to OVA sensitization, and then the mice were sensitized and challenged with OVA as indicated in Fig. 1A. Samples were collected and analyzed 24 h after the last challenge. The results indicated that IL-28B was stably overexpressed in serum and BALF for at least 17 days (Fig. 1B and C). Importantly, compared with control group, overexpression of IL-28B significantly inhibited OVA-induced allergic asthma as evidenced by the marked decrease in the numbers of eosinophils, neutrophils and lymphocytes as well as IgE production and airway resistance in the mouse lung (Fig. 1D–H). Consistently, the reduced leukocyte infiltration was also observed in the peribronchial areas (Fig. 1I). In addition, overexpression of IL-28B greatly suppressed OVA-induced goblet cell hyperplasia and mucus hypersecretion in airway epithelium (Fig. 1J). These data suggest that IL-28B plays an inhibitory role in OVA-induced airway inflammation.

### Overexpression of IL-28B increased the number and frequency of NK cells in OVA-challenged mouse lungs

**Figure 2:**
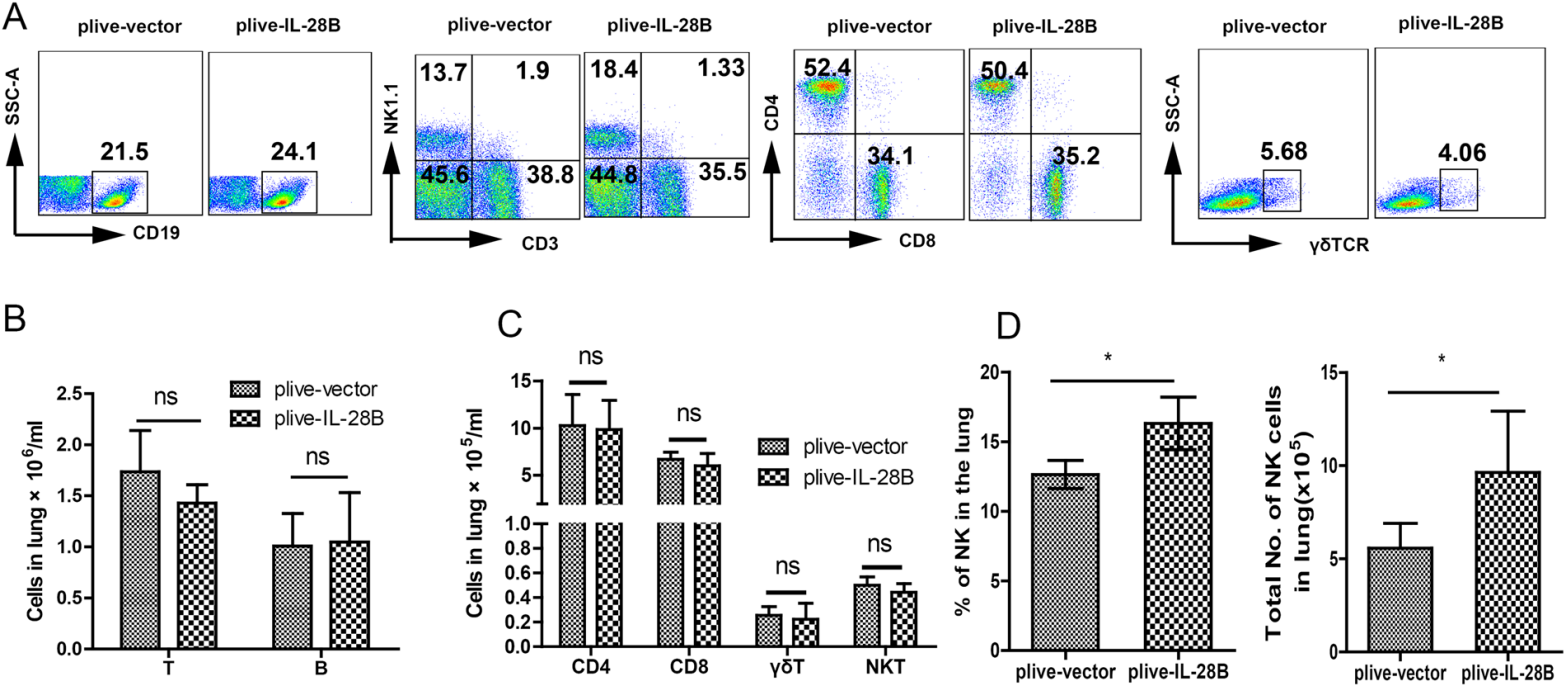
IL-28B increased the number and abundance of NK cells in lungs of asthmatic mice. (**A–C**) Flow cytometry (**A**) and cell counting assays (**B** and **C**) for frequency and numbers of lymphocyte subsets in the lungs of asthmatic mice. ns: non-significant. (**D**) Cell counting assay for frequency and number of NK cells in the lungs of asthmatic mice. *p < 0.05; ns: non-significant (*n* = 5 mice per group). Results shown are representative of three independent experiments.

In order to determine the mechanisms whereby IL-28B inhibited OVA-induced airway inflammation, we examined the numbers of lymphocytes in IL-28B-overexpressed lungs after OVA challenge. The results showed that IL-28B had no effects on the numbers of T (CD4+, CD8+) cells, B cells, NKT cells γδT cells (Fig. 2A–C) as well as macrophages and Dendritic cells (Fig. S1A,B and D), but significantly increased the number and percentage of NK cells in OVA-challenged lungs (Fig. 2D), suggesting the possible involvement of NK cells in inhibiting OVA-induced airway inflammation.

### NK cells mediated IL-28B-suppressed airway inflammation

**Figure 3:**
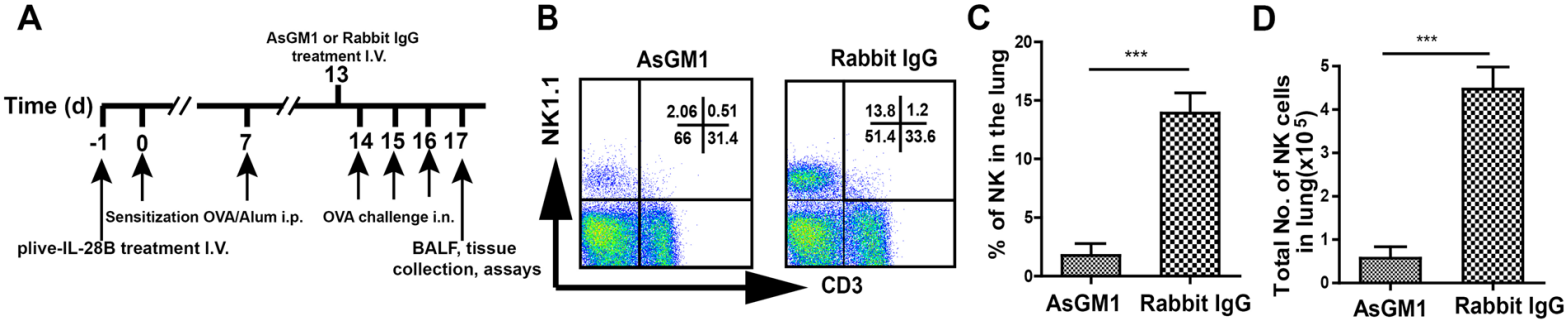
NK cells were depleted in mice by AsGM1. (**A**) Schematic timeline for AsGM1 antibody administration to OVA-sensitized mice and Rabbit IgG was used as control. (**B–D**) Flow cytometry (**B**) and cell counting assays for abundance (**C**) and numbers (**D**) of NK cells in the lung. AsGM1 is anti-asialo ganglioside-monosialic acid. ***p < 0.001 (n = 4 mice per group). Results shown are representative of three independent experiments.

**Figure 4:**
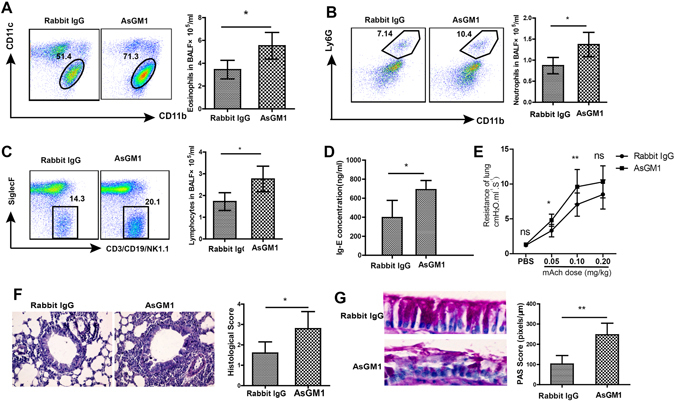
NK cells were essential for IL-28B-suppressed airway inflammation. (**A–C**) Flow cytometry and cell counting assays for abundance and numbers of eosinophils (**A**), neutrophils (**B**) and lymphocytes (**C**) in BALF. (**D**) The effect of AsGM1 treatment on IgE production. (**E**) The effect of AsGM1 treatment on the airway resistance in the mouse lung. (**F**) Histological assessment of lung inflammation in rabbit IgG- and AsGM1-treated asthmatic mice. (**G**) PAS staining for mucus production in lung epithelium of rabbit IgG- and AsGMA1-treated asthmatic mice. *p < 0.05; **p < 0.01; ns: non-significant (*n* = 5 mice per group). Results shown are representative of three independent experiments.

In order to determine if NK cells are involved in IL-28-suppressed airway inflammation, NK cells were depleted in mice by intraperitoneal injections of 50 µl AsGM1 antibody one day prior to the initial OVA challenge, and rabbit IgG was used as control (Fig. 3A). As shown in Fig. 3B–D, AsGM1 antibody effectively depleted NK cells for at least 4 days, but has no significant effect of anti-ASGM1 treatment on the numbers of Macrophages and Dendritic cells (Fig. S1A,C and E), suggesting the high specificity of anti-ASGM1 treatment. Flow cytometry and cell counting assays indicated that AsGM1 injection significantly increased the frequency and numbers of eosinophils, neutrophils, and lymphocytes in BALF as well as IgE production and airway resistance in the mouse lung compared with control group (Fig. 4A–E), as well as greatly promoting the leukocyte infiltration in the peribronchial areas (Fig. 4F). Furthermore, AsGM1 injection markedly enhanced IL-28B-suppressed goblet cell hyperplasia and mucus hypersecretion in airway epithelium (Fig. 4G). These data demonstrate that IL-28B inhibition of OVA-induced airway inflammation is NK cell-dependent.

### IL-28B inhibition of OVA-induced airway inflammation depended on IFN-γ

**Figure 5:**
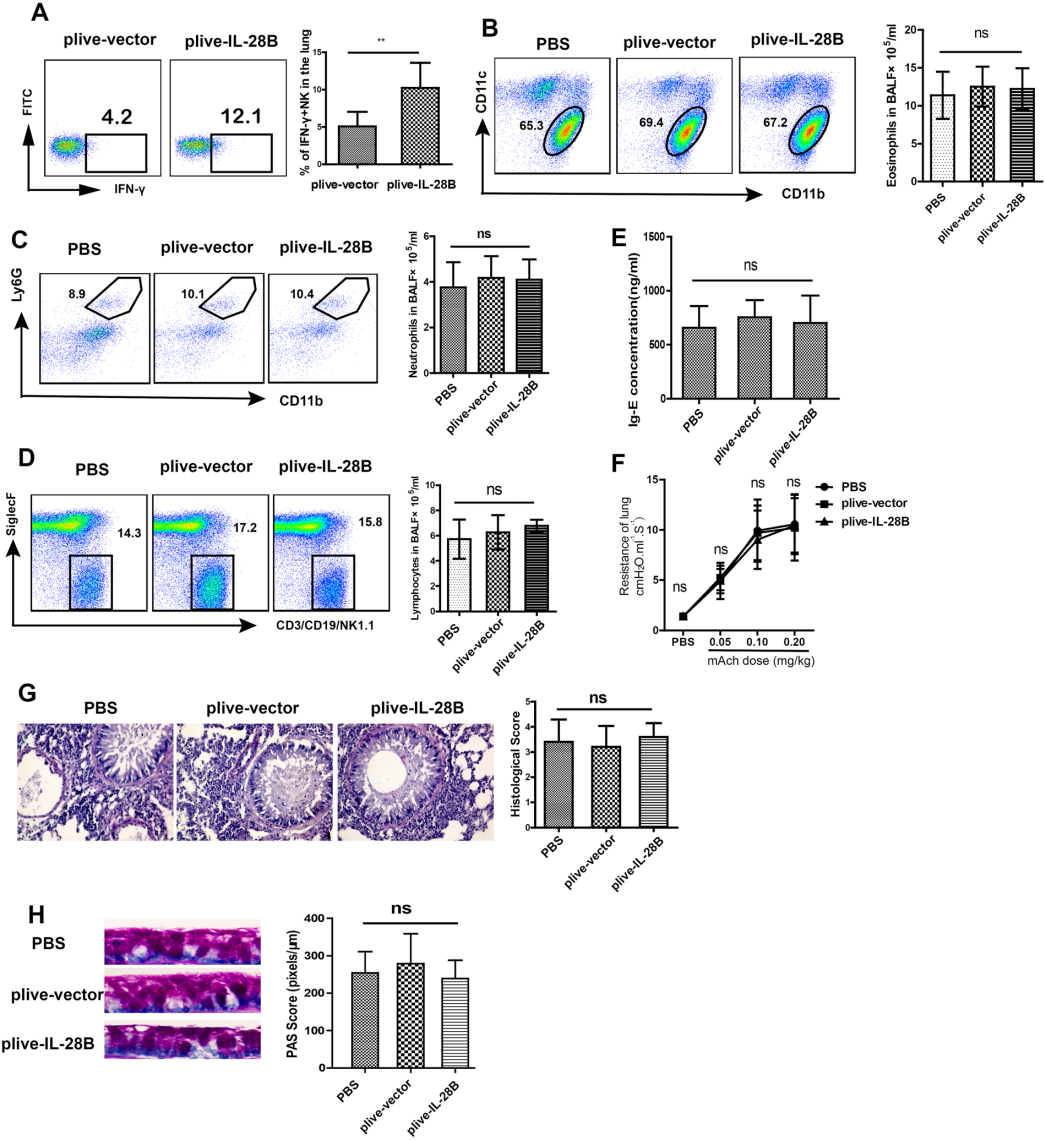
IFN-γ was essential for IL-28B-suppressed airway inflammation. (**A**) Flow cytometry assay for abundance of IFN-γ-producing NK cells in lungs of asthmatic WT mice. **p < 0.01 (*n* = 10 mice); (**B–D**) Flow cytometry and cell counting assays for abundance and numbers of eosinophils (**B**) neutrophils (**C**) and lymphocytes (**D**) in BALF of IFN-γ−/− mice treated with plive-vector- or plive-IL28B. (**E**) The effect of AsGM1 treatment on IgE production. (**F**) The effect of plive-IL-28B on the airway resistance in the mouse lung. (**G**) Histological assessment of lung inflammation in IFN-γ−/− mice treated with PBS, plive-vector or plive-IL28B. H, PAS staining for mucus production in lung epithelium of IFN-γ−/− mice treated with PBS, plive-vector or plive-IL28B. ns: non-significant (*n* = 10 mice). Results shown are representative of three independent experiments.

In order to further investigate the mechanism by which NK cells mediate IL-28B-inhibited airway inflammation induced by OVA, NK cell-produced cytokines were examined. The results showed that IL-28B overexpression resulted in significant increase in the number of IFN-γ-producing NK cells (Fig. 5A). IFN-γ is an important cytokine that inhibits Th2-mediated inflammatory response^25^, suggesting that IFN-γ might mediate IL-28B- or NK cell-inhibited Th2 inflammatory response^26^. We overexpressed IL-28B in IFN-γ−/− mice by tail vein injections followed by OVA sensitization and challenge, and found IL-28B overexpression had no effects on the numbers of eosinophils, neutrophils, and lymphocytes in BALF as well as IgE production and airway resistance in the mouse lung compared with control plasmid or PBS group (Fig. 5B–F). Consistent with this finding, there was no significant changes in the degree of leukocyte infiltration into the peribronchial areas, airway goblet cell hyperplasia and mucus hypersecretion (Fig. 5G,H). These data demonstrate that IFN-γ is essential for IL-28B-suppressed airway inflammation.

## Discussion

IFN- λ, as a member of IFN family, includes IL-28A, IL-28B and IL-29. It regulates both innate and adaptive immunity, and has potential antiviral and anticancer properties^27^. IL-28A can inhibit allergic airway inflammation through regulating DC function^16^. However, the role of IL-28B in inhibiting allergic airway inflammation remains unknown. In the present study, we demonstrate that IL-28B can suppress OVA-induced allergic airway inflammation via promoting NK cell polarization.

NK cells are a distinct type of innate lymphocytes analogous to the cytotoxic T cells of the adaptive immune systems. They play a critical role in combating allergen- or virus-induced respiratory diseases like asthma^28^. IL-28 acts directly on NK cells that express IL-28R to regulate NK cell effector functions and enhance IFN-γ production^20, 27^. IFN-γ-producing NK cells then enter local lymph nodes to interact with DCs, leading to DC activation and IL-27 secretion. IL-27 further induces NK cell to produce IFN-γ which inhibits Th2 and Th17 polarization, through a positive feedback mechanism^29, 30^. NK cell subsets NK1 and NK2 have been found to differently regulate expression of immunity-related genes like NK receptor and IgE, during inflammatory processes^31^.

In this study, we indicate that IL-28B overexpression effectively decreases the frequencies and numbers of inflammatory effector cells including eosinophils, neutrophils and lymphocytes in BALF in OVA-induced mouse asthma model (Fig. 1). IL-28B appears to modulate the activation of pulmonary epithelium that also expresses IL-28R, because IL-28B overexpression reduces airway goblet cell hyperplasia and subsequent mucus production (Fig. 1). Interestingly, IL-28B exerts no effects on the frequencies and numbers of T and B cells in lungs of asthmatic mice, suggesting that other cell type is involved in IL-28B inhibition of the OVA-induced airway inflammation. Our data show that NK cells may be the right ones as evidenced by IL-28B-induced increases in frequency and number of NK cells, especially those of IFN-γ-producing NK1 cells (Figs. 2,3 and 4). NK1 cell polarization that increases IFN-γ secretion probably play an important role in IL-28B-suppressed airway inflammation. Indeed, both NK cell depletion by ASGM1 and IFN-γ knockout reverses the inhibitory effect of IL-28B on OVA-induced allergic asthma (Figs. 4 and 5). Our findings are consistent with the previous studies that IFN-γ is responsible for allergic airway inflammation and mucus secretion^32, 33^. In addition, patients with asthma exhibit reduced NK cell-mediated IFN-γ production which weakens the capabilities of promoting DC maturation and killing immature DCs, whereas NK2 cell frequency is enhanced^34–36^, indicating the possible involvement of NK cells in human asthma. These findings are further confirmed by our present study in a mouse model of OVA-induced allergic asthma.

However, the true roles of NK cells in the regulation of airway inflammation still remain controversial. It was reported that NK cell depletion *in vivo* abrogated development of allergic airway inflammation^37^. The opposite functions of NK cells in allergic airway diseases may be likely due to the different experimental conditions such as strength and duration of allergen sensitization and challenge, which may lead to the diverse NK cell activities in inflammatory responses.

In summary, our study identifies IL-28B as a novel negative regulator of OVA-induced allergic airway inflammation like asthma, suggesting a new role of IL-28B in adaptive immunity. Therefore, IL-28B may serve as a potential therapeutic agent for effective intervention of allergic asthma via promoting NK1 cell polarization and IFN-γ production.

## Electronic supplementary material

Supplementary Information

## References

[1] Lambrecht BN, Hammad H (2015). The immunology of asthma. Nat Immunol.

[2] Royce SG (2014). Mechanistic insights into the contribution of epithelial damage to airway remodeling. Novel therapeutic targets for asthma. Am J Respir Cell Mol Biol.

[3] Lunding L, Wegmann M (2015). NK cells in asthma exacerbation. Oncotarget.

[4] Wang J (2012). Lung natural killer cells in mice: phenotype and response to respiratory infection. Immunology.

[5] Peng H, Wisse E, Tian Z (2016). Liver natural killer cells: subsets and roles in liver immunity. Cell Mol Immunol.

[6] Pandya AD (2011). Identification of human NK17/NK1 cells. PLoS One.

[7] Loza MJ, Perussia B (2001). Final steps of natural killer cell maturation: a model for type 1-type 2 differentiation?. Nat Immunol.

[8] Katsumoto T (2004). STAT6-dependent differentiation and production of IL-5 and IL-13 in murine NK2 cells. J Immunol.

[9] Lang PA (2012). Natural killer cell activation enhances immune pathology and promotes chronic infection by limiting CD8+ T-cell immunity. Proceedings of the National Academy of Sciences of the United States of America.

[10] Morandi B, Bougras G, Muller WA, Ferlazzo G, Munz C (2006). NK cells of human secondary lymphoid tissues enhance T cell polarization via IFN-gamma secretion. Eur J Immunol.

[11] Wei H (2005). Involvement of human natural killer cells in asthma pathogenesis: natural killer 2 cells in type 2 cytokine predominance. J Allergy Clin Immunol.

[12] Ozdemir, O. Type 2 natural killer cells in asthma? *J Allergy Clin Immunol* **116**, 1165-1166, author reply 1166–1167, doi:10.1016/j.jaci.2005.08.007 (2005). 10.1016/j.jaci.2005.08.007 16275396

[13] Di Santo JP (2008). Natural killer cells: diversity in search of a niche. Nat Immunol.

[14] Karimi K, Forsythe P (2013). Natural killer cells in asthma. Frontiers in immunology.

[15] Lasfar A (2006). Characterization of the mouse IFN-lambda ligand-receptor system: IFN-lambdas exhibit antitumor activity against B16 melanoma. Cancer Res.

[16] Koltsida O (2011). IL-28A (IFN-lambda2) modulates lung DC function to promote Th1 immune skewing and suppress allergic airway disease. EMBO Mol Med.

[17] Sheppard P (2003). IL-28, IL-29 and their class II cytokine receptor IL-28R. Nat Immunol.

[18] Doyle SE (2006). Interleukin-29 uses a type 1 interferon-like program to promote antiviral responses in human hepatocytes. Hepatology (Baltimore, Md.).

[19] Mennechet FJ, Uze G (2006). Interferon-lambda-treated dendritic cells specifically induce proliferation of FOXP3-expressing suppressor T cells. Blood.

[20] Souza-Fonseca-Guimaraes F (2015). NK cells require IL-28R for optimal *in vivo* activity. Proceedings of the National Academy of Sciences of the United States of America.

[21] Li F, Zhu H, Sun R, Wei H, Tian Z (2012). Natural killer cells are involved in acute lung immune injury caused by respiratory syncytial virus infection. Journal of virology.

[22] Tong S (2017). Natural killer cell activation contributes to hepatitis B viral control in a mouse model. Sci Rep.

[23] Tian J, Zhu T, Liu J, Guo Z, Cao X (2015). Platelets promote allergic asthma through the expression of CD154. Cell Mol Immunol.

[24] Edwards MR, Johnston SL (2011). Interferon-lambda as a new approach for treatment of allergic asthma?. EMBO Mol Med.

[25] Kujur W, Gurram RK, Haleem N, Maurya SK, Agrewala JN (2015). Caerulomycin A inhibits Th2 cell activity: a possible role in the management of asthma. Sci Rep.

[26] Liu Y (2017). Uncompromised NK cell activation is essential for virus-specific CTL activity during acute influenza virus infection. Cell Mol Immunol.

[27] Lazear HM, Nice TJ, Diamond MS (2015). Interferon-lambda: Immune Functions at Barrier Surfaces and Beyond. Immunity.

[28] Erten G, Aktas E, Deniz G (2008). Natural killer cells in allergic inflammation. Chemical immunology and allergy.

[29] Fekete T, Koncz G, Szabo B, Gregus A, Rajnavolgyi E (2017). Interferon gamma boosts the nucleotide oligomerization domain 2-mediated signaling pathway in human dendritic cells in an X-linked inhibitor of apoptosis protein and mammalian target of rapamycin-dependent manner. Cell Mol Immunol.

[30] Chong WP (2015). NK-DC crosstalk controls the autopathogenic Th17 response through an innate IFN-gamma-IL-27 axis. J Exp Med.

[31] Aktas E (2005). Different natural killer (NK) receptor expression and immunoglobulin E (IgE) regulation by NK1 and NK2 cells. Clinical and experimental immunology.

[32] Durrant DM, Gaffen SL, Riesenfeld EP, Irvin CG, Metzger DW (2009). Development of allergen-induced airway inflammation in the absence of T-bet regulation is dependent on IL-17. J Immunol.

[33] Cohn L, Homer RJ, Niu N, Bottomly K (1999). T helper 1 cells and interferon gamma regulate allergic airway inflammation and mucus production. J Exp Med.

[34] Scordamaglia F (2008). Perturbations of natural killer cell regulatory functions in respiratory allergic diseases. J Allergy Clin Immunol.

[35] Forsythe P, Inman MD, Bienenstock J (2007). Oral treatment with live Lactobacillus reuteri inhibits the allergic airway response in mice. Am J Respir Crit Care Med.

[36] Kawashima T (2011). Lactobacillus plantarum strain YU from fermented foods activates Th1 and protective immune responses. Int Immunopharmacol.

[37] Haworth O, Cernadas M, Levy BD (2011). NK cells are effectors for resolvin E1 in the timely resolution of allergic airway inflammation. J Immunol.

