# Supplementary material for: Interleukin-28B dampens airway inflammation through up-regulation of natural killer cell-derived IFN-γ
Source: Sci Rep. 2017 Jun 15;7:3556. doi: 10.1038/s41598-017-03856-w (PMC5472588; doi:10.1038/s41598-017-03856-w)
Supplement: Supplementary file 1 — Supplementary Information [file 41598_2017_3856_MOESM1_ESM.pdf]

Supplementary information

Interleukin-28B dampens airway inflammation through up-regulation of natural killer cell-derived IFN-γ

Bailing Yan[1], Feng Chen[2], Lijun Xu[3*], Yanshi Wang[4] and Xuefu Wang[4,5*]

[1]Emergency Department, the First Hospital of Jilin University, Changchun, 130021, China.

[2]Dermatology Department, China-Japan Union Hospital of Jilin University, Changchun 130033, China

[3]Department of Respiratory Medicine, the First Hospital of Jilin University, Changchun, 130021, China

[4]Institute of Immunology, School of Life Sciences, University of Science and Technology of China, Hefei, Anhui, 230027, China

[5]School of Pharmacology, Anhui Medical University, Hefei, Anhui, 230032, China;

*Corresponding authors：

Lijun Xu: Tel: +86 431 84808365. E-mail addresses: lijunxu001@sina.com.

Xuefu Wang: Tel: +86 13721051707. E-mail addresses: wangxuefu@ustc.edu.cn

Supplementary Figures and Legends

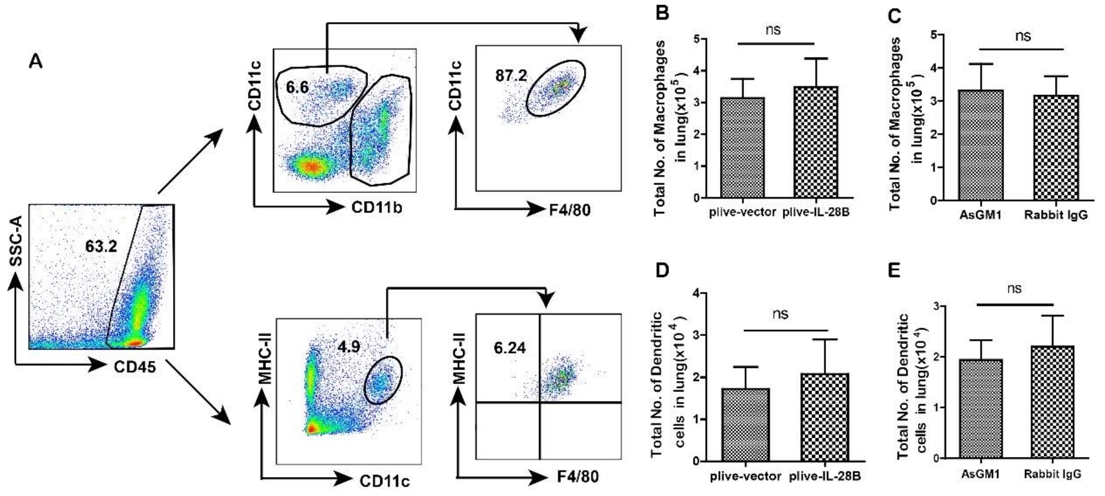

Fig.S1 Anti-ASGM1 and plive-IL-28B treatment have no significant effect on the number of macrophages and dendritic cells of the lung. A, Gating strategy for the detection of macrophages and dendritic cells by flow cytometry. B and D, The effects of plive-IL-28B treatment on the numbers of macrophages (B) and dendritic cells (D). C and E, The effects of Anti-ASGM1 treatment on the numbers of macrophages (C) and dendritic cells (E). ns: significant difference. Results shown are representative of three independent experiments.
